# Detailed Mechanisms Underlying Neutrophil Bactericidal Activity against *Streptococcus pneumoniae*

**DOI:** 10.3390/biomedicines11082252

**Published:** 2023-08-11

**Authors:** Zachary Tsai, Kyle A. Carver, Henry H. Gong, Kosuke Kosai, Jane C. Deng, Matthew J. Worley

**Affiliations:** 1 Division of Pulmonary and Critical Care Medicine, University of Michigan School of Medicine, Ann Arbor, MI 48105, USAkycarver@med.umich.edu (K.A.C.); k-kosai@nagasaki-u.ac.jp (K.K.); worleyma@med.umich.edu (M.J.W.); 2 Research Service and Pulmonary Section, Veterans Affairs Ann Arbor Healthcare System, Ann Arbor, MI 48105, USA; gonghy@umich.edu; 3 Department of Laboratory Medicine, Nagasaki University Graduate School of Biomedical Sciences, Nagasaki 852-8523, Japan

**Keywords:** bactericidal, degranulation, innate immunity, neutrophil extracellular trap, neutrophil, phagocytosis, reactive oxygen species, *Streptococcus pneumoniae*, Toll-like receptor

## Abstract

Neutrophils are an essential cellular component of innate immunity and control bacterial infections through a combination of intracellular and extracellular killing methods. Although the importance of neutrophils has been established, the exact methods used to handle particular bacterial challenges and the efficiency of bacterial killing remain not well understood. In this study, we addressed how neutrophils eliminate *Streptococcus pneumoniae* (*Spn*), a leading cause of community acquired and post-influenza bacterial pneumonia. We analyzed killing methods with variable bacterial:neutrophil concentrations and following priming with PAM3CSK4 (P3CSK), an agonist for Toll-like-receptor 2 (TLR2). Our results show that murine neutrophils display surprisingly weak bactericidal activity against *Spn*, employing a predominantly extracellular mode of killing at lower concentrations of bacteria, whereas challenges with higher bacterial numbers induce both extracellular and intracellular elimination modes but require TLR2 activation. TLR2 activation increased reactive oxygen species (ROS) and neutrophil extracellular trap (NET) formation in response to *Spn*. Despite this, supernatants from P3CSK-stimulated neutrophils failed to independently alter bacterial replication. Our study reveals that unstimulated neutrophils are capable of eliminating bacteria only at lower concentrations via extracellular killing methods, whereas TLR2 activation primes neutrophil-mediated killing using both intracellular and extracellular methods under higher bacterial burdens.

## 1. Introduction

*Streptococcus pneumoniae* (*Spn*) is the leading cause of bacterial pneumonia in adults [1]. Despite the availability of antibiotics and multiple anti-pneumococcal vaccines, pneumococcal pneumonia continues to cause significant morbidity and mortality [1]. Antibiotic resistance, particularly the emergence of multidrug resistant bacterial strains, have highlighted the need for complementary therapies that can enhance host defense mechanisms [2]. This is of particular importance in patient populations who may exhibit various forms of immunosuppression, such as aged patients, patients with advanced chronic organ dysfunction (e.g., end-stage liver disease), or survivors of persistent critical illness [3]. Neutrophils are the dominant cellular component of innate immune response and play a critical role in mediating responses to bacterial, fungal and viral pathogens [4,5,6]. They comprise the early and most abundant immune effector cell population at sites of bacterial infections [7]. Unfortunately, despite the long-standing recognition that neutrophils are critical for eliminating bacterial pathogens, understanding the optimal conditions for neutrophil killing of bacterial pathogens is still rudimentary, thus limiting our ability to design therapies aimed at enhancing neutrophil function.

Patients that have neutropenia (<1500 neutrophils/microliter) have increased susceptibility to bacterial infections, and thus, immunotherapy aimed at augmenting host defense has largely focused on increasing neutrophil numbers [8]. Despite extensive examination of neutrophil function, therapies have not been able to fully harness the therapeutic potential of neutrophils in patients with infectious diseases. The ratio of pathogens to neutrophils has been investigated in vitro and may provide insights into the defects seen in neutropenic patients. In vitro studies using *Staphylococcus aureus* demonstrated that ratios of bacteria to neutrophils above 1:1 strongly decrease phagocytotic rate of neutrophils [9], while other research has suggested that neutrophil-mediated bacterial killing was concentration dependent with only ratios below 0.1:1 of *Spn*:neutrophils showing killing [10]. In stark contrast, Li et al. noted that neutrophil concentration rather than bacteria to neutrophil ratio was critical for effective bacterial killing [11]. New therapeutics that correct neutropenia while also maintaining or enhancing neutrophil functions may prove helpful in treating patients with bacterial pneumonia.

Toll-like receptors (TLRs) expressed by neutrophils have been shown to have significant effects on neutrophil function via the modulation of RNA profiles following stimulation [12]. TLR2 is a lipoprotein receptor that plays an integral for recognizing gram-positive bacteria, including *Spn* [13,14,15]. TLR2 activation primes neutrophils allowing for increased cytokine production, superoxide production, and increased phagocytosis [12,16]. Bacterial killing by neutrophils can occur either intracellularly or extracellularly. Intracellular killing requires the phagocytosis of the bacteria and killing with reactive oxygen species (ROS) or antimicrobial proteins from granules, particularly when pathogens are opsonized with antibodies or complement [17,18,19,20]. Extracellular killing relies on degranulation of antimicrobial proteins or the formation of neutrophil extracellular traps (NETs) [19]. NETs, however, have proven to be controversial in their ability to kill pathogens with reports of limited killing [19,21,22], while others report potent killing and reduction in virulence factors [23,24,25]. Although evidence supports the improved intracellular neutrophil killing with the activation of TLR2, little is known about whether extracellular killing plays a role [26]. Understanding how TLR2 regulates intracellular and extracellular killing mechanisms could provide valuable insights into how immune priming modulates the strategies neutrophils employ to eliminate bacteria.

We conducted an in-depth examination of the factors underlying effective intracellular and extracellular mediated neutrophils killing of *Spn*, which is the most common cause of community acquired pneumonia and secondary pneumonia following influenza. We report that, surprisingly, neutrophils are poorly bactericidal against *Spn*. We examined what aspects of neutrophil bactericidal activity are effective at different neutrophil to bacteria ratios, analyzed the effects of TLR2 activation on neutrophil bactericidal ability, and investigated mechanisms by which TLR2 activation altered intracellular and extracellular killing of *Spn*. Additionally, we demonstrated that robust ROS and NET release following PAM3CSK (P3CSK) stimulation of TLR2 was enhanced by co-culture with *Spn*.

## 2. Materials and Methods

### 2.1. Streptococcus Pneumoniae Culture

*Spn* serotype 23 was used for all experiments. *Spn* was grown for 5 h in Todd Hewitt broth at 37 °C, 5% CO_2_, then pelleted at 1250 g for 10 min at room temperature (RT). The bacterial pellet was resuspended in phosphate buffered saline (PBS) and optical density measured at 600 nm to determine an estimated CFU/mL (colony forming units/mL) based on an established growth curve. Serial dilutions were cultured overnight on sheep blood agar plates to confirm the correct bacteria CFU for multiplicity of infections (MOIs) ratios.

### 2.2. Bacterial Opsonization

Bacteria was opsonized using Innovative Research (Novi, MI, USA) mouse complement (Cat #IMSC57BL6COMPL5ML). Bacterial cells were incubated in a 37 °C incubator with 5% CO_2_ for 30 min before use.

### 2.3. MPRO Cells Culture and Differentiation

The Murine promyelocytic (MPRO) cell line can be differentiated into mature murine neutrophils that have similar levels of secondary granule formation, respiratory burst and phagocytic capacity comparable to mouse peripheral blood neutrophils [27,28]. The MPRO cells were generously supplied by Peter Gaines at University of Massachusetts, Lowell. MPRO cells were cultured in AIM-V media (Gibco, Thermo Fisher Scientific, New York, NY, USA cat# A3830801) supplemented with 5% fetal bovine serum and 10 ng/mL GM-CSF (PeproTech, Thermo Fisher Scientific, New York, NY, USA, cat# 315-03). MPRO cells were differentiated into mature neutrophils with 10 µM all-trans-Retinoic acid (ATRA) (Sigma-Aldrich, St. Louis, MO, USA, cat# R2625) for 5 days. Cell differentiation was validated by Ly6G expression via flow cytometry and cellular morphology by Cytospin and Diff-Quick staining, consistent with published studies [27,29]. Prior to use, the cells were washed to remove the differentiation media and resuspended in RPMI.

### 2.4. Primary Murine Neutrophils Isolation

Primary murine neutrophils were obtained from bone marrow of C57BL6 mice from our in-house colony at the Ann Arbor Veterans Affairs Medical Center. All experiments were approved by the Veterans Affairs Institutional Animal Care and Use Committee and were performed in accordance with NIH guidelines. The neutrophils were isolated per published methods [30]. Briefly, mice were euthanized by CO_2_ and femur and tibia bones were harvested. Muscle was removed, the bones were rinsed in 70% ethanol and PBS prior to cutting off the ends of each bone. RPMI with 5% fetal bovine serum was used to flush through the bone to remove the marrow. A single-cell suspension was then strained through a 70 μm cell strainer followed by red blood cell lysis. Neutrophils were then isolated using the EasySep PE positive selection kit (STEMCELL Technologies, Vancouver, Canada 17666) using PE-conjugated anti-Ly6G antibody (clone: 1A8, BioLegend, San Diego, CA, USA).

### 2.5. Phagocytosis Assay and Bactericidal Assay

Phagocytosis and bactericidal assays were conducted on differentiated MPROs and primary mouse neutrophils. MPROs had 200,000 cells in 25 μL per well and primary mouse neutrophils were seeded at 100,000 cells in 25 μL per well in V-bottom 96 well-plates. Cells were stimulated with TLR2 ligand, PAM3CSK4 (P3CSK, InvivoGen cat# tlrl-pms), at 50 μg/mL for 60 min at 37 °C and 5% CO_2_.

Subsequently, opsonized *Spn* (high MOI > 0.2, low MOI < 0.01) was added to P3CSK-stimulated and unstimulated neutrophils for 30 min. To assess intracellular killing, at 30 min after the addition of bacteria, plates were spun down for 1 min at 300 g at RT. Supernatants were collected at this time point for enumeration of extracellular bacteria for the extracellular killing assay below. The wells with the pelleted cells were washed twice with RPMI to remove extracellular bacteria. A set of wells were immediately lysed with pH 11 sterile water for 5 min to release intracellular bacteria. Serial dilutions of cell lysates were plated on blood agar plates to determine intracellular bacterial CFU (i.e., at 30 min). The remaining wells with the washed cells were further incubated in serum-free RPMI for an additional 60 min (37 °C, 5% CO_2_). At 90 min, cells were washed and lysed as above to enumerate intracellular bacterial CFU. Intracellular bactericidal activity was determined by determining how many bacterial CFU were present in the cell lysates obtained at 90 min compared to 30 min.

For the extracellular killing assay, run concurrently, a separate set of wells with *Spn* and unwashed neutrophils were incubated for 90 min total. Supernatants obtained at 30 min (as described above) were serially diluted and plated to determine extracellular bacterial number. At 90 min, cells were spun down as above, and CFU of supernatants obtained at 90 min compared to bacterial CFU in supernatants collected at 30 min.

### 2.6. ROS Generation via CM-H2DCFDA

CM-H2DCFDA cleavage was utilized to determine the ROS generated by neutrophils in response to *Spn* exposure at different MOI. Primary neutrophils were resuspended in HBSS with CM-H2DCFDA at a final concentration of 5 μM and seeded at 100,000 neutrophils per well in 96-well black-walled clear bottom plates. Cells were cultured with or without stimulation with 50 μg/mL of P3CSK in the presence or absence of *Spn* at high or low MOI. The fluorescent signal was measured using the bottom detector of a fluorescence plate reader (BioTek Synergy H1), 493/522 nm, and Gen5 software version 3.11. The plate was immediately read for a 0 min time point and then every 5 min for the first hour, followed by measurements every 15 min for 16 h. The index relative fluorescent units (iRFU) was calculated as follows: iRFU = absolute maximum RFU of test sample/absolute maximum of reference sample, where the reference samples were unstimulated and without *Spn* as per previously published methods [31,32].

### 2.7. NETs Release

Isolated primary neutrophils were resuspended in HBSS (Gibco, Cat# 14025092) and seeded at 50,000 cells per well into 96-well black-walled clear bottom plates (Fisher, Hampton, NH, USA, cat#07-200-565). The neutrophils were stained with Sytox green (Invitrogen, Waltham, MA, USA, cat # S7020) at a final concentration of 5 μM. The neutrophils were then cultured with or without 50 μg/mL of P3CSK for 60 min before the additional of *Spn* bacteria at high or low MOI. The fluorescent signal was measured using the bottom detector of a fluorescence plate reader (BioTek Synergy H1), 493/534 ex/em, and Gen5 software. An initial (0 min time point) measurement was taken immediately after Sytox green and P3CSK were added to the neutrophils, with measurements taken every 30 min thereafter for 13 h. The index relative fluorescent units (iRFU) were calculated as above.

### 2.8. Assessment of Bactericidal Activity of Neutrophil Supernatants

Primary neutrophils at a concentration of 1 × 10^6^ cells/mL were incubated in HBSS (Gibco cat# 14025092) or stimulated with 50 μg/mL P3CSK in HBSS for 60 min. Cells were then centrifuged at 500 g for 5 min at RT and supernatants collected. Bacteria at high MOI or low MOI were cultured in 100 µL of either unstimulated or stimulated supernatants. Bacteria were incubated for 90 min at 37 °C with 5% CO_2_, then serial diluted and plated out for bacterial CFU enumeration.

### 2.9. Statistical Analysis

Prism 9.3.1 (GraphPad Software, San Diego, CA, USA) was used to calculate significance using either Mann–Whitney U tests when two samples were compared, and Kruskal–Wallis nonparametric one-way ANOVA adjusted for multiple comparisons where applicable.

## 3. Results

### 3.1. MPRO Killing of Extracellular but Not Intracellular Spn Is Dependent upon Bacteria:Neutrophil Ratio

**Figure 1:**
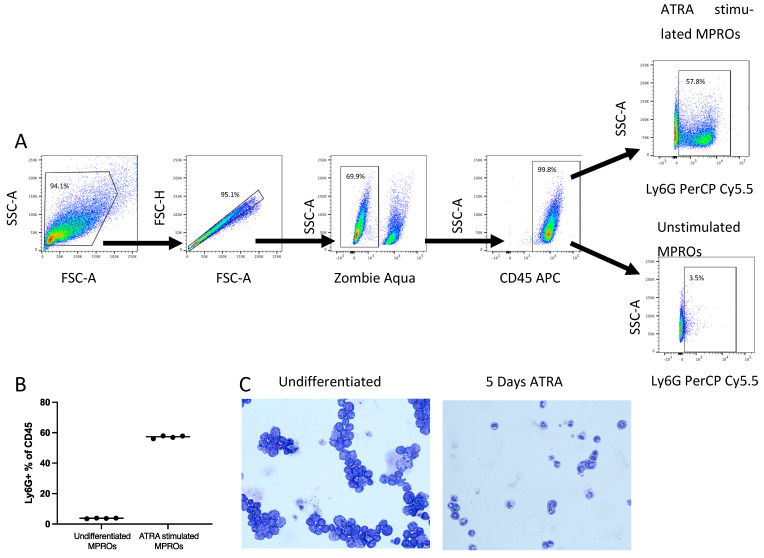
Validation of MPRO differentiation following ATRA stimulation for 5 days. At day 5 following ATRA stimulation cells were stained for flow cytometry and the gating strategy presented in (**A**) was used to determine the Ly6G expression on CD45 positive cells (**B**). Diff-Quick staining of MPROs differentiation following ATRA stimulation for 5 days show distinct morphology changes (**C**).

**Figure 2:**
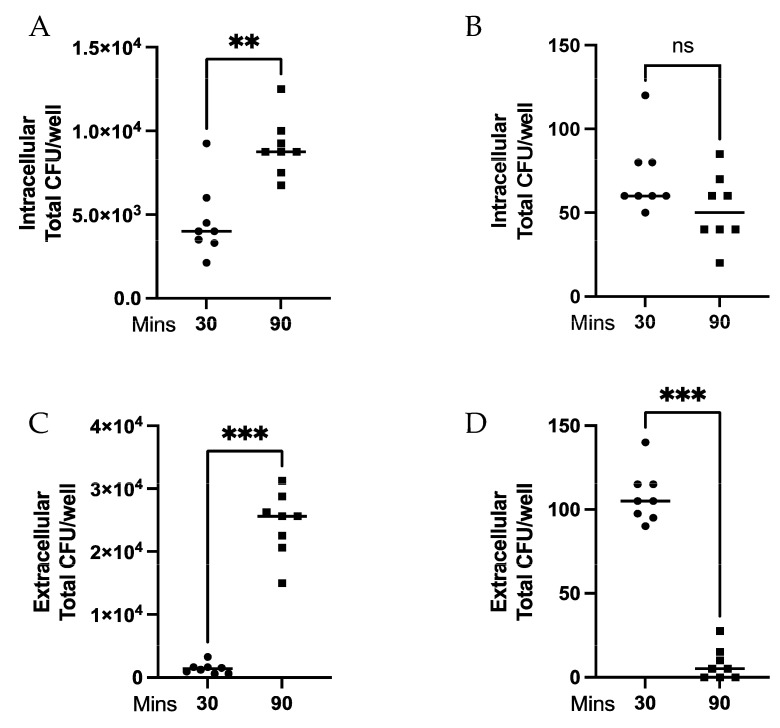
Intracellular and extracellular killing of *Spn* by MPROs. MPROs were differentiated for 5 days until mature and then incubated with *Spn* at a high MOI for (**A**,**C**) and low MOI for (**B**,**D**). The intracellular (**A**,**B**) and extracellular (**C**,**D**) killing of *Spn* by neutrophil was measured lysing the neutrophils and by collecting the supernatants and measuring remaining CFU. Significant differences are denoted ** *p* < 0.01, *** *p* < 0.001, ns *p* > 0.05, data are representative of two replicates.

The ratio of neutrophils to bacteria has been reported as a potential mediator for efficient phagocytosis [10]. We investigated if neutrophils displayed differential abilities to kill bacteria or limit bacterial division depending on ratio of bacteria to neutrophils. MPROs were differentiated for 5 days with ATRA, with differentiation validated by flow cytometry (Figure 1A,B) and cytospins (Figure 1C) prior to use. Differentiated MPROs were co-cultured for 30 min and 90 min with high or low MOIs of *Spn* and the intracellular and extracellular bacteria killing was evaluated. There were significantly (*p* = 0.0031) more bacteria intracellularly at 90 min compared to 30 min at high MOI (Figure 2A) demonstrating inefficient intracellular killing. Alternatively, at the low MOI there was no difference in intracellular bacteria (Figure 2B). The bacteria present in the supernatant was significantly higher (*p* = 0.0002) at 90 min compared to 30 min at high MOI (Figure 2C); however, this was reversed at the low MOI (*p* = 0.0002) (Figure 2D). Our data indicate that there is greater extracellular killing by neutrophils at low MOI with containment and limited bacteria division that is not possible at the high MOI. This demonstrates that there is a *Spn*:neutrophil ratio dependent killing of the bacteria and it is primarily mediated through extracellular mechanisms.

### 3.2. Ability of MPROs to Kill a Greater Bacterial Challenge, Intra- and Extracellularly, Is Dependent upon TLR2 Activation

**Figure 3:**
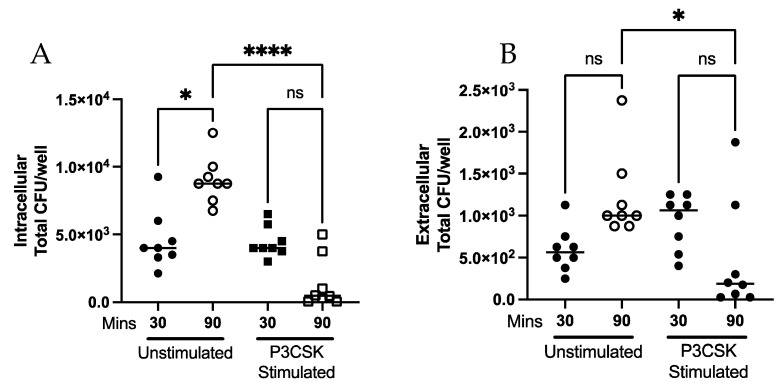
Intracellular and extracellular killing of *Spn* by MPROs following TLR stimulation. MPROs were differentiated for 5 days until mature and cultured with or without P3CSK stimulation (60 min) prior to adding in *Spn* at a high MOI and the intracellular (**A**) and extracellular (**B**) killing assessed. Intracellular and extracellular *Spn* was measured at 30 and 90 min following addition of bacteria. Significant differences are denoted * *p* < 0.05, **** *p* < 0.0001, ns *p* > 0.05, data are representative of two replicates.

Given that our initial data showed that neutrophils were not effective at killing phagocytosed *Spn* at low or high MOI (Figure 2A,B), we next wanted to determine the effect of prior TLR2 activation on intracellular and extracellular killing of *Spn*. *Spn* was incubated at a high MOI with neutrophils that were pre-stimulated with P3CSK or without stimulation. There was a significant decrease (*p* ≤ 0.0001) in intracellular (phagocytosed) *Spn* following 90 min of culture in the P3CSK-stimulated compared to the unstimulated neutrophils (Figure 3A). There was significantly (*p* = 0.0144) less extracellular *Spn* with P3CSK stimulation compared to unstimulated cultures at 90 min (Figure 3B). Our data indicate that P3CSK stimulation results in improved intracellular and extracellular killing of bacteria by neutrophils.

### 3.3. Ability of Primary Mouse Neutrophils to Contain Intracellular and Kill Extracellular Bacteria Is Dependent upon TLR2 Activation

**Figure 4:**
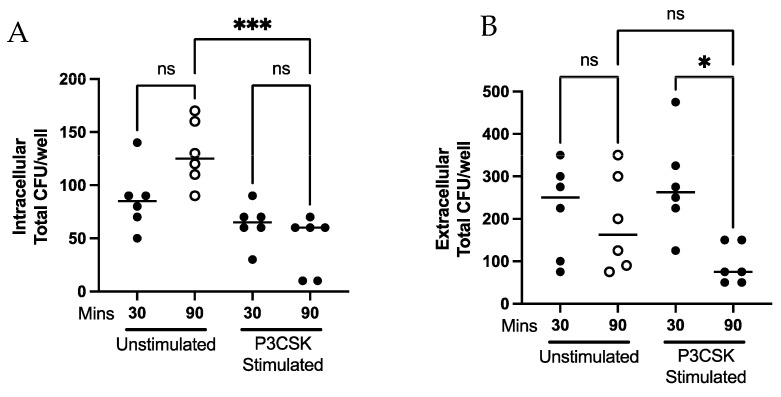
Intracellular and extracellular killing of *Spn* by primary mouse bone marrow neutrophils following TLR2 stimulation. Primary bone marrow neutrophils were isolated and cultured with or without P3CSK stimulation (60 min) prior to adding in *Spn* at a low MOI and the intracellular (**A**) and extracellular (**B**) killing assessed. Samples were collected at 30 and 90 min following the addition of *Spn* to the neutrophils and the presence of intracellular and extracellular *Spn* assessed. Significant differences are denoted * *p* < 0.05, *** *p* < 0.001, ns *p* > 0.05, data are representative of two replicates.

To validate the findings in differentiated MPRO cells, primary mouse neutrophils were evaluated for phagocytosis and extracellular killing activity. Bone marrow neutrophils were isolated and cultured with and without P3CSK stimulation prior to incubation with a low MOI of *Spn*. There was a significant decrease (*p* = 0.0009) in intracellular bacteria following 90 min of co-culture of P3CSK-stimulated neutrophils compared to unstimulated (Figure 4A). P3CSK-stimulated cells showed a significant amount of extracellular killing (*p* = 0.0170) when comparing 30 to 90 min, which were unaltered in the unstimulated samples (Figure 4B). The data show a nonsignificant trend towards an increase in extracellular killing by P3CSK-stimulated cells at 90 min compared to unstimulated controls. Thus, the enhanced intracellular and extracellular killing displayed by P3CSK stimulation of MPRO cells were replicated in primary mouse neutrophils. This lends support to MPROs as a model for murine neutrophils for stimulated killing. More importantly, the data show activation of TLR2 increases containment of *Spn* by phagocytosis and enhancement of extracellular killing.

### 3.4. Primary Mouse Neutrophils Induce ROS Differently When Exposed to Low vs. High MOI of Spn

**Figure 5:**
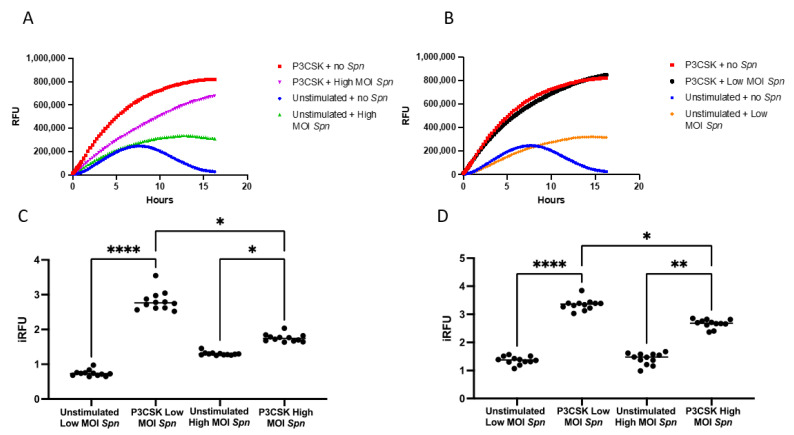
Reactive oxygen species (ROS) response by primary mouse bone marrow neutrophils with and without TLR2 stimulation in the presence of *Spn*. Primary bone marrow neutrophils were isolated and cultured with and without P3CSK stimulation in the presence of *CM-H2DCFDA* and *Spn* at high (**A**) and low (**B**) MOI. The relative fluorescent intensity of *CM-H2DCFDA* was measured on a fluorescent plate reader every 5 min for the first hour and then every 15 min following for 6 h. The indexed RFU (iRFU) was then calculated to give the fold change in the reference sample for either the first 3 h (**C**) or the full 16 h (**D**) time course. Significant differences are denoted * *p* < 0.05, ** *p* < 0.01, **** *p* < 0.0001.

Neutrophils are able to employ a number of mechanisms to kill bacterial pathogens, with many of them utilizing ROS. To examine the relative contribution of TLR2 stimulation and exposure to *Spn* towards induction of ROS, we measured ROS responses over time with CM-H2DCFDA in primary mouse neutrophils with and without stimulation with P3CSK and co-cultured with high and low MOI of *Spn* for 16 h. The P3CSK-stimulated neutrophils with no *Spn* induced greater amounts of ROS than the P3CSK-stimulated neutrophils with high MOI of *Spn* (Figure 5A). Unstimulated neutrophils with no *Spn* developed some ROS responses reaching a peak at 7–8 h then decreased. The P3CSK-stimulated neutrophils in the presence of a high MOI of *Spn* responses continued to increase to the last measurement at 16 h (Figure 5A).

Interestingly, the P3CSK-stimulated neutrophils showed similar levels of ROS to the P3CSK-stimulated neutrophils cultured with low MOI of *Spn* (Figure 5B). The unstimulated neutrophils co-cultured with a low MOI of *Spn* followed the same trend as the high MOI (Figure 5A,B). To evaluate the statistical relevance of these kinetic curves, we calculated the iRFU for the first 3 h and for the full kinetic curve. In the first 3 h, the P3CSK-stimulated neutrophils with a low MOI of *Spn* was almost four-fold higher (*p* ≤ 0.0001) than the unstimulated and low MOI of *Spn* (Figure 5C). Although the difference was less pronounced, there was still a significant increase in iRFU in the high MOI of *Spn* with P3CSK compared to without P3CSK (*p* = 0.0358). There was a significant increase (over two-fold) in the P3CSK-stimulated neutrophils with low MOI of *Spn* compared to high MOI of *Spn* (*p* = 0.0358). Over the full kinetic curves these differences are maintained; however, the differences had increased between the unstimulated vs P3CSK with a high MOI of *Spn* (*p* = 0.0043; Figure 5D). This suggests that P3CSK promotes a sustained production of ROS by neutrophils, whereas the maximum ROS generation appears to be delayed in the presence of high MOI of *Spn*.

### 3.5. Primary Mouse Neutrophils Induce NETs Differently When Exposed to Low vs. High MOI of Spn

**Figure 6:**
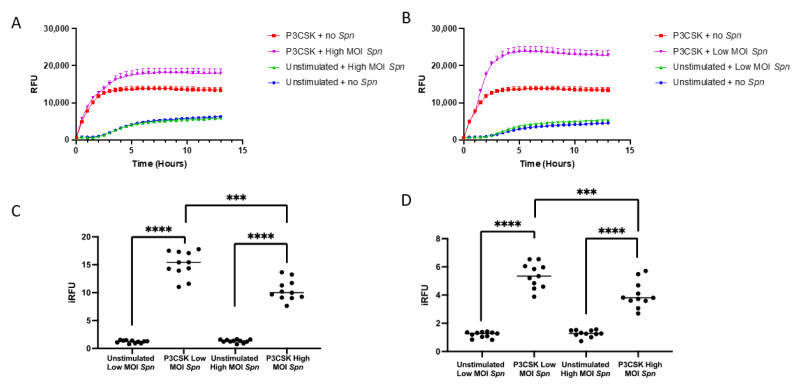
Kinetic measure of NET release by primary mouse bone marrow neutrophils with and without TLR2 stimulation in the presence of *Spn*. Primary bone marrow neutrophils were isolated and cultured with and without P3CSK stimulation in the presence of Sytox green for 1 h and then *Spn* at high (**A**) and low (**B**) MOI was added. The relative fluorescent intensity of sytox green was measured on a fluorescent plate reader every 30 min for 13 h. The indexed RFU (iRFU) was then calculated to give the fold change in the reference sample for either the first 3 h (**C**) or the full 13 h (**D**) time course. Significant differences are denoted *** *p* < 0.001, **** *p* < 0.0001, data are representative of two replicates.

Much of the prior research on the role that TLR2 plays in neutrophil activation has evaluated the effects on phagocytosis [33,34,35,36]. Therefore, the role that NETs play in controlling bacteria following activation of TLR2 has not been well investigated. We investigated if primary bone marrow neutrophils with prior stimulation by P3CSK were able to generate NETs in response to low or high MOI of *Spn* by measuring the magnitude of Sytox green fluorescence kinetically over 13 h. In the absence of P3CSK stimulation, similar levels of NETs were produced between neutrophils without *Spn* and at a high or low MOI of *Spn* (Figure 6A,B). This indicates that *Spn* is unable to induce NET release from unstimulated neutrophils. Conversely, P3CSK-stimulated neutrophils induce NET release rapidly, but the addition of high MOI *Spn* increases the maximum levels detected (Figure 6A). This trend was recapitulated in the low MOI of *Spn* (Figure 6B). The maximum responses of NET release were higher in the low MOI with P3CSK than with the high MOI responses, which is consistent with earlier results showing a difference in extracellular killing.

To evaluate the statistical significance of the NET kinetic curves, we calculated the iRFU for the first 3 h and for the full kinetic curve. During the first 3 h, the combination of P3CSK and low MOI of *Spn* induced 12-fold higher (*p* ≤ 0.0001) amounts of NETs than unstimulated and low MOI of *Spn* alone (Figure 6C). There was a significant increase (eight-fold) in NETs in the high MOI of *Spn* with P3CSK compared to without P3CSK stimulation (*p* ≤ 0.0001). Low MOI of Spn resulted in a significant increase in P3CSK-stimulated neutrophils compared to high MOI (*p* = 0.0001) (Figure 6C). Over the full kinetic curves these differences are maintained, with differences in the P3CSK-stimulated neutrophils between low MOI *Spn* compared to high MOI *Spn* remaining highly significant (*p* ≤ 0.0001; Figure 6D). The maximum differences in NET generation appears to occur within the first few hours as highlighted by the higher fold changes observed within the first 3 h (Figure 6C) compared to over the full course of NET formations (Figure 6D). This may be due to longer term culture of bone marrow neutrophils that may start to spontaneously form NETs at later time points lessening the fold changes from the reference samples.

### 3.6. Extracellular Killing Is Not Mediated via Soluble Factors

**Figure 7:**
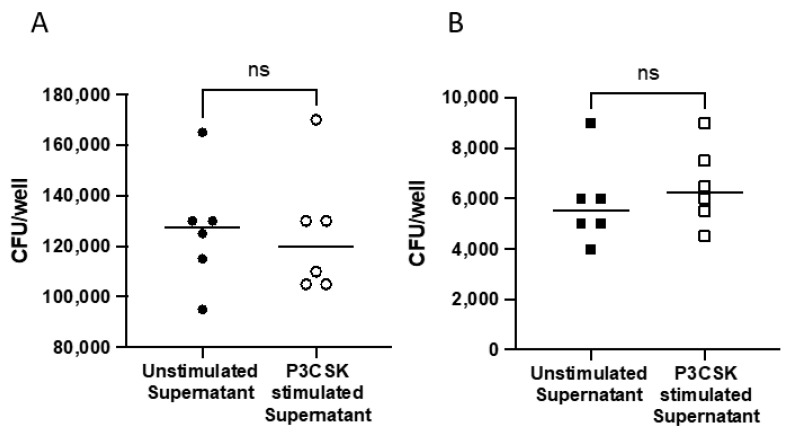
Supernatants from P3CSK-stimulated primary mouse bone marrow neutrophils did not inhibit *Spn* growth. Primary mouse bone marrow neutrophils were cultured with or without P3CSK in the absence of bacteria for 60 min and supernatants collected. *Spn* was cultured for 90 min in the supernatants at either a high (**A**) or low (**B**) MOI relative to the original neutrophil stimulation. The data are representative of two replicates. ns *p* > 0.05.

Neutrophils are able to release a number of soluble factors to mediate antibacterial responses. To determine if soluble factors released from neutrophils were leading to increased extracellular killing, we next examined the bactericidal activity of primary mouse neutrophil supernatants following P3CSK stimulation. The supernatants of stimulated and unstimulated neutrophils were collected fresh and cultured with a low or high MOI of *Spn*. There was no significant bacterial growth inhibition at either the high (Figure 7A) or low MOI (Figure 7B). The data suggest that stimulated neutrophils are killing bacteria extracellularly using another bactericidal method aside from soluble granule release, or that neutrophils require direct interaction with bacteria to mediate these responses.

## 4. Discussion

Given the critical role of neutrophils in host innate immunity, there is a need for an improved mechanistic understanding in what governs the effectiveness of their responses. We investigated how bacteria density impacts the ability of neutrophils to mediate intracellular and extracellular killing. We demonstrated that neutrophils differentiated from MPROs are able to kill extracellularly in a bacteria concentration dependent manner and that stimulation via TLR2 increases killing by both intracellular and extracellular methods. This result was validated in primary bone marrow neutrophils. At a low MOI of *Spn,* there was greater enhancement of both ROS and NET release following stimulation. Despite the ROS generation and NET release, the soluble factors contained in stimulated supernatants were unable to inhibit *Spn* replication in the absence of cells.

There are two current concepts underlying our understanding of important factors governing neutrophil antibacterial activity–one being the ratio of neutrophils to bacteria, and the other that neutrophil concentration are critical for effective bacterial killing by neutrophils. Esposito et al. observed concentration dependent killing with MOI at or below 0.1 *Spn*:neutrophils [10], while, Li et al. showed that neutrophil concentration (300,000–400,000 neutrophils/mL) was critical for effective bacterial killing rather than MOI [11]. Neither of these studies made the distinction between intracellular and extracellular bacterial killing by neutrophils. Our results showed that unstimulated MPROs mediated significant extracellular killing at a low MOI of *Spn* (Figure 2). These results in part support the observation of Esposito et al.; however, this was only observed in extracellular killing. Despite our neutrophil concentration far exceeding the critical concentration reported by Li et al., we did not observe significant intracellular killing regardless of MOI. This may be a product of Li et al. completing their study with primary human neutrophils and a different bacterial species. Further work will need to be performed to directly compare mouse neutrophils and human neutrophils in the intracellular and extracellular killing of *Spn* versus other clinically important bacteria. Nonetheless, our results generate a more detailed mechanistic understanding of an important aspect of neutrophil antimicrobial function, and provide a framework by which neutrophil antibacterial function from human patients can be comprehensively evaluated. Furthermore, given that in clinical settings it has long been assessed the adequacy of neutrophil responses purely in terms of having sufficient neutrophil numbers, our studies highlight the importance of understanding the complexity underlying the deceptively simple concept of “effective” neutrophil-mediated bacterial killing.

TLR2 stimulation has been shown to trigger or prime neutrophils for cytokine release, superoxide generation and increased phagocytosis [12]. Our results in MPROs and primary mouse neutrophils build upon these studies, in that we observe that TLR2 stimulation increases intracellular and extracellular killing of *Spn* (Figure 3 and Figure 4). Due to the increase in the rate of intracellular killing depicted in Figure 3A and Figure 4A, this could account for fewer bacteria remaining extracellularly which would lessen the extracellular burden, thereby potentiating extracellular killing. Despite the increases in extracellular killing observed following P3CSK stimulation in Figure 3B and Figure 4B, supernatants from stimulated cells failed to inhibit *Spn* growth in Figure 7A,B. This suggests that the stimulated neutrophils must have direct interaction with *Spn* following stimulation to be able to kill the bacteria, as the soluble factors secreted by neutrophil degranulation was unable to limit the bacterial growth (Figure 7A,B). Further studies would be needed to fully elucidate the extracellular mechanisms that TLR2-stimulated neutrophils are employing to control *Spn*. However, the data presented in Figure 6 highlight that TLR2-stimulated neutrophils can rapidly release NETs, which were enhanced by exposure to *Spn* and were unlikely to have been collected in the supernatants evaluated in Figure 7.

NETs have emerged over the last two decades as critical tools in the arsenal of responses that neutrophils can employ to limit bacterial infections [37]. It has previously been established that the activation of TLR2 can lead to the formation of NETs [38,39,40]. Our results suggest extracellular killing of *Spn* with NETs is enhanced with prior TLR2 stimulation of neutrophils (Figure 4B). The NETs produced by neutrophils are also enhanced in the presence of a low MOI of *Spn* compared to a high MOI (Figure 6A,B). Thus, the abundance of pathogens may lead to differences in how neutrophils prioritize different antimicrobial responses. In a fungal model of NET release, it was shown that neutrophils are able to sense the size of the pathogen and can alter the responses mediated [4]. The role that NETs play in controlling bacterial infection is complex and not fully elucidated. NETs have been shown to directly kill bacteria and degrade virulence factors. In addition, NETs may act as a barrier and limit spread of bacterial infection. NETs have also been shown to increase in phagocytic uptake of bacteria by other phagocytes and potentially neutrophils [13]. The wrapping of bacteria by NETs is thought to facilitate greater killing and phagocytosis. In this study, we observed the increase in NET release was associated with decreased bacterial replication but did not investigate the other potential antibacterial properties of NETs. Further studies would need to be completed to determine the relative degree to which any of these alternative antimicrobial functions of NETs are regulated by TLR2 and magnitude of bacterial stimulation.

In summary, TLR2 stimulation of neutrophils is able to enhance neutrophils to kill more *Spn*, both intracellularly and extracellularly. At a low MOI compared to a high MOI of *Spn,* we observe a greater amount of ROS generation and NET release by neutrophils. Soluble factors released by TLR2-stimulated neutrophils were unable to inhibit *Spn* growth, demonstrating there is a need for direct interaction between neutrophils and *Spn* to allow for extracellular killing, which we postulate is likely a result of NET release. Further research should investigate if these findings apply to other bacteria and other *Spn* strains.

## Data Availability

The original contributions presented in the study are included in the article. Further inquiries can be directed to the corresponding author.

## Abbreviations

RFU: relative fluorescent units; CFU, colony forming units; MOI, multiplicity of infections; *Spn*, *Streptococcus pneumoniae*; NETs, neutrophil extracellular traps; ROS, reactive oxygen species; P3CSK, PAM3CSK; TLR2, Toll-like receptor 2; iRFU, indexed relative fluorescent units.
